# Post intubation tracheal stenosis

**DOI:** 10.4103/0972-5229.45081

**Published:** 2008

**Authors:** Sajal De, Sarmishtha De

**Affiliations:** **From:** Department of Pulmonary Medicine, Bhopal Memorial Hospital and Research Centre, Bhopal, India; 1 Department of ENT, People's Medical College, Bhopal, India

**Keywords:** Balloon bronchoplasty, post intubation tracheal injury, web like stenosis

## Abstract

Tracheal stenosis following prolonged intubation is a relatively rare but a serious problem. However, some degree of airway injury is common following intubation, no matter whether it is prolonged or of short duration. Here, we are reporting a fifty six year old male patient who developed multiple web like tracheal stenosis following intubation with high volume low pressure cuff endotracheal tube. Subsequently, the stenosis was successfully dilated by balloon bronchoplasty.

## Introduction

The cuff-pressure of endotracheal tubes play an important role on the development of tracheal damage. To minimize this injury, use of high volume and low pressure cuff endotracheal tubes are advocated. Surgical resection for the management of post intubation tracheal stenosis remains a controversial issue because of the risk of recurrence at the site of anastomosis and also these patients are often at higher surgical risk. Other alternative therapies like laser resection, stent placement and balloon bronchoplasty can also be tried. Laser resection, stent placement are expensive and require expertise and available only at tertiary care centres. Balloon bronchoplasty is a relatively simple procedure, which can be done under sedation and even at the bedside.

## Case Report

A fifty six year old gentleman was referred to our hospital with the complaints of gradually increasing difficulty in breathing and dry cough for one month and an audible wheeze for the last seven days. He denied any history of fever, weight loss or anorexia. His background history revealed that he was a smoker and had quit smoking three months earlier, and was taking antihypertensive medicines for the last three years. Three months prior to the present complaints, the patient had received thrombolytic therapy (Urokinase) for an inferior wall-plus right ventricular myocardial infarction. Few hours after thrombolysis, he had developed severe cardiogenic shock with left ventricular dysfunction and was put on mechanical ventilatory support, and also needed an intra aortic balloon pump to support his heart. Subsequently, he underwent angiography and PTCA to the left circumflex coronary artery. The patient made a good recovery after definitve treatment for heart failure was initiated and was extubated four days later and was discharged from the unit in a stable condition after twelve days.

**Figure 1:**
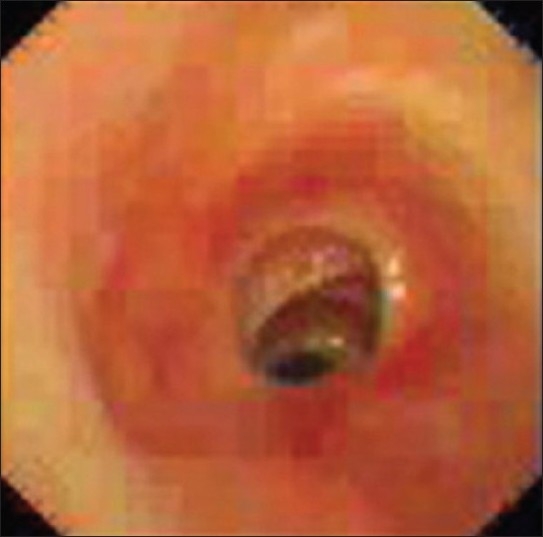
Bronchoscopic image showing multiple web like stenosis

The patient's general physical examination was unremarkable except for an audible wheeze. A Chest radiograph was normal. Pulmonary function tests showed reduction in the expiratory flow rate. Direct laryngoscopy showed normal movement of both the vocal cords. He underwent diagnostic bronchoscopy and on bronchoscopy, multiple membranous web like stenosis on the upper tracheal cartilages with small pedunculated soft tissue growth distal to the stenosis was seen [Figure 1]. Punch biopsy was taken from the growth and histopathological examination showed chronic granulation tissue comprising of numerous thick wall blood vessels with dense stroma and reactive fibroblasts.

**Figure 2:**
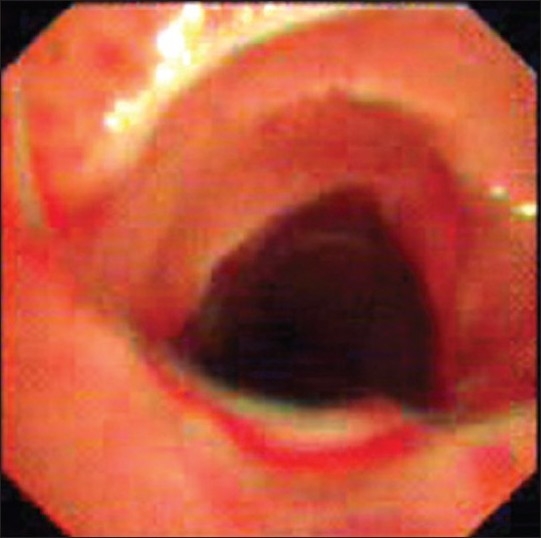
Bronchoscopic image after successful dilation

Under short general anesthesia, the granulation tissue was removed by electrosurgery and the stenosed segment was dilated by PPD™ esophageal progressive balloon Dilator (TeleMed System Inc. USA, Balloon length 8 cm and largest external diameter of 17mm with 118 PSI). The maximum diameter was held for 30s and the procedure was repeated thrice. The patent was hyperoxygenated before each maneuver. After the third dilation, the achieved diameter was as per expectation [Figure 2].

After the procedure, the patient received prednisolone 30mg once daily and Amoxy-clavulanic acid combination 625mg twice daily for three days. After three months, the follow up bronchoscopy showed no further narrowing and the patient remains asymptomatic for the last one year.

## Discussion

In earlier days, post intubation tracheal stenosis was one of the common complications of prolonged intubation. With the introduction of endotracheal tubes with a large area of contact (high volume, low pressure cuff) the incidence of post intubation tracheal stenosis in intensive care units has remarkably reduced. However, post intubation stenosis still remains an important cause of acquired tracheal obstruction. When the cuff pressure exceeds the mucosal capillary pressure (30 mm of Hg) of the trachea, the mucosa that lies between the cuff of the balloon and the underlying cartilages develops ischemia. Long standing ischemia can leads to ulceration and chondritis of tracheal cartilages, followed by fibrotic healing, leading to progressive tracheal stenosis. One prospective study had shown even intubation with high volume low pressure cuffed tubes, 11% of critically ill patients had developed tracheal stenosis at the cuff site.[1] Usual factors responsible for stenosis are: cuff pressure, size of the tube relative to the tracheal lumen, duration of intubation, cardiovascular status during intubation, movement of tube during the period of intubation, sex and age of the patient, material from which cuff is manufactured and the possible adverse effects of steroids etc.[2] However, tracheal stenosis can also be developed by intubation lasting as short as 24 hours only.[3]

These patients may remain asymptomatic for a variable period and then develop difficulty in expectoration and dyspnea on exertion and can progress to airway obstruction with the development of a stridor. Post intubation tracheal stenosis is often misdiagnosed as asthma and is not diagnosed at initial presentation in as many as 44% of patients.[4] Patients usually remain asymptomatic until the trachea has stenosed to 30% of its original diameter, and it may take as long as three months before the diagnosis.[4]

During spirometry, flow-volume loops exhibit a characteristic reduction in peak expiratory flow, with a plateau in the expiratory curve. However, a classical loop cannot be seen unless the diameter is narrowed to 8-10mm. Spirometry results are often complicated by concomitant lung diseases and it is not a reliable diagnostic technique. Chest X-ray rarely detects stenosis but a CT scan will provide precise information regarding exact location, extent of stenosis and the nature of surrounding soft tissues.

Bronchoscopy is the mainstay of diagnosis and it also rules out other diseases (i.e. vocal cord palsy, tracheomalacia). A simple bronchoscopic procedure for tracheal stenosis had been developed by Freitag *et al,*[5] for the classification of tracheobronchial stenosis and to compare the results and analyze the outcome over a wide range of interventions. They divided tracheal stenosis into structural or functional types and further classified it by the degree of stenosis, location and the transition zone. Post intubation web like tracheal stenosis is an example of Type 4 stenosis.

The various options for treating tracheal stenosis are dilation, laser resection, stenting and resection anastomosis. Brichet *et al*,[6] reviewed 32 consecutive cases of post intubation tracheal stenosis at their institution and proposed rigid bronchoscopy with neodymium ±yttrium aluminium garnet (Nd-YAG) laser resection or stent implantation (removable stent) as the first-line of treatment. In their study, laser resection was curative in 66% of web-like stenosis. In patients with complex stenosis or failed laser treatment (up to three sessions) removable stents were inserted. Subsequently, if the patient was judged operable, the stent was removed and the patient underwent definitive surgery. The serious complications of laser therapy are perforation of major intrathoracic blood vessels, pneumothorax or pneumomediastinum secondary to perforation of the airway wall and endobronchial ignition.

Tracheal sleeve resection is the definitive surgical treatment. The stenotic segments are resected and end to end anastomosis are done. Alternatively, various synthetic materials can be used to bridge the gap. However, sleeve resection can only be possible for patients with good neurological, cardiovascular and respiratory condition. Usual contraindications for surgery are, a possibility of the requirement for prolonged ventilatory support in the post operative period, medical contraindications and a long length of stenosis which is not technically feasible for resection and anastomosis. The failure rate of surgery is about 15%. In the series of 340 patients reported by Bonette *et al,*[7] definitive cure was obtained at the first attempt in 265 patients, after a second tracheal resection in six patients.

Balloon bronchoplasty can be done via a rigid or flexible bronchoscope with or without fluoroscopic guidance. A guide wire can be inserted first through the working channel of a bronchoscope, and a balloon is passed over the guide wire, and the guidewire later removed. Alternatively, the balloon can be passed by the side of the bronchoscope. The length of the balloon should be sufficient to reach at least one cm below and above the stenosed segment. The balloon can be filled with saline or dilute contrast medium and inflation can be done by a pressure syringe device to achieve the specified pressure. The balloon should stretch 2-3 mm beyond the desired diameter. The balloon can be kept inflated for 15-150 seconds and there is no such definitive study for the adequate dilation time. The patients should be hyperventilated in between the procedure to maintain the oxygen saturation above 90%, during the procedure. The mechanism of balloon bronchoplasty is expanding the tracheal wall by creating a longitudinal split on posterior tracheal wall. Balloon bronchoplasty is an easy and effective way to relieve both the proximal and distal airway stenosis and can also be repeated. Those patients who require more than one balloon dilation may need additional therapies i.e. laser or stent placement. The overall complication rates are as less as 5%. Balloon bronchoplasty can often cause superficial and deep ulceration of the tracheal mucosa, which usually heals without much granulation tissue or fibrosis. If the tracheal cartilages are not damaged, a good outcome can be achieved by a single dilation. Excessive balloon inflation may cause rupture of the airway leading to hemorrhage, pneumothorax, pneumomediastinum, or mediastinitis.

Mayse *et al,*[8] evaluated the efficiency and safety of balloon bronchoplasty in 26 patients. Balloon dilation as the only mode of therapy was used in 26% of their cases and when used as a part of a multimodal approach, the success rate was 100%. No significant adverse events were observed. For benign non inflammatory stenosis and annular cicatrical stenosis, the success rate of balloon bronchoplasty is high. Long term efficacy of balloon bronchoplasty in benign stenosis after 32 months is reported as 43%.[9]

The systemic use of corticosteroids or antibiotics are probably unnecessary, although no definitive data is available.

In spite of the use of a high volume and low pressure cuffed endotracheal tube, our patient developed post intubation tracheal stenosis within 4 days of intubation, possibly due to underlying hypotension. As more and more patients receive prolonged mechanical ventilatory support in ICU or undergo a tracheostomy for ventilatory support, the risk of developing stenosis remains high. Tracheal damage and subsequent stenosis can occur in any patient after intubation of any duration and a high index of suspicion is required to diagnose these cases at the earliest. Tracheal stenosis should be treated with Laser therapy or by surgical resection. However, Laser therapy is not readily available because of the prohibitory cost and the surgical management is often not possible because of associated risk factors.

Balloon bronchoplasty is an inexpensive procedure. The disposable esophageal balloon dilator is sufficient for the procedure, cost of which is approximately INR 6500 and can be reused. The other advantages are: it can be done at the bedside, can be repeated and short term improvement can sustain the patient for other definitive and complex management. So balloon bronchoplasty should be offered as the initial treatment for post intubation web like stenosis, especially in the developing countries.
